# Homocysteine and Mitochondria in Cardiovascular and Cerebrovascular Systems

**DOI:** 10.3390/ijms21207698

**Published:** 2020-10-18

**Authors:** Peter Kaplan, Zuzana Tatarkova, Monika Kmetova Sivonova, Peter Racay, Jan Lehotsky

**Affiliations:** Department of Medical Biochemistry, Jessenius Faculty of Medicine, Comenius University in Bratislava, Mala Hora 4D, 036 01 Martin, Slovakia; Zuzana.Tatarkova@uniba.sk (Z.T.); Monika.Kmetova.Sivonova@uniba.sk (M.K.S.); Peter.Racay@uniba.sk (P.R.); Jan.Lehotsky@uniba.sk (J.L.)

**Keywords:** homocysteine, hyperhomocysteinemia, mitochondria, heart, brain, oxidative stress, ROS

## Abstract

Elevated concentration of homocysteine (Hcy) in the blood plasma, hyperhomocysteinemia (HHcy), has been implicated in various disorders, including cardiovascular and neurodegenerative diseases. Accumulating evidence indicates that pathophysiology of these diseases is linked with mitochondrial dysfunction. In this review, we discuss the current knowledge concerning the effects of HHcy on mitochondrial homeostasis, including energy metabolism, mitochondrial apoptotic pathway, and mitochondrial dynamics. The recent studies suggest that the interaction between Hcy and mitochondria is complex, and reactive oxygen species (ROS) are possible mediators of Hcy effects. We focus on mechanisms contributing to HHcy-associated oxidative stress, such as sources of ROS generation and alterations in antioxidant defense resulting from altered gene expression and post-translational modifications of proteins. Moreover, we discuss some recent findings suggesting that HHcy may have beneficial effects on mitochondrial ROS homeostasis and antioxidant defense. A better understanding of complex mechanisms through which Hcy affects mitochondrial functions could contribute to the development of more specific therapeutic strategies targeted at HHcy-associated disorders.

## 1. Introduction

**Figure 1:**
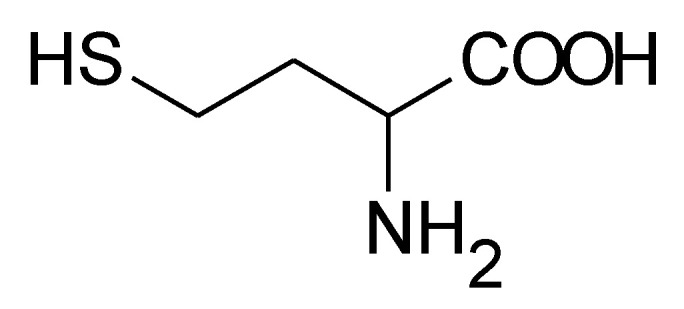
Structural formula of homocysteine (M_r_ = 135.2).

Homocysteine (Hcy) is a sulfur-containing amino acid (Figure 1) formed during metabolism of methionine, an essential amino acid derived from dietary proteins. The metabolic pathway involved in Hcy formation is important due to formation of S-adenosylmethionine (SAM), a source of methyl group for methylation reactions, such as DNA methylation or formation of catecholamines. S-adenosylhomocysteine (SAH), which is formed from SAM after transfer of methyl group to various substrates, is hydrolyzed by SAH hydrolase to Hcy and adenosine. Released Hcy can be remethylated to methionine via folate and vitamin B_12_-dependent reaction and/or catabolized to amino acid cysteine by the vitamin B_6_-dependent pathway. Deficiencies in vitamins and/or enzymes involved in Hcy metabolism as well as some pathological conditions result in elevated blood plasma concentrations of Hcy, called hyperhomocysteinemia (HHcy). Based on fasting Hcy concentrations, HHcy may be classified as mild (15–30 μmol/L), intermediate (30–100 μmol/L), and severe (>100 μmol/L) [1]. Severe HHcy occurs as a result of genetically determined deficiencies of enzymes involved in Hcy, B_12_, and folate metabolism and is manifested by mental retardation, osteoporosis, vascular damage, and other complications [2]. Although severe HHcy is rare, mild or intermediate elevations of Hcy are common in the general population, and their major causes seem to be less severe genetic mutations and variations and deficiencies in folate, vitamin B_6_, and vitamin B_12_. Mild/intermediate HHcy was shown to be an independent risk factor for many disorders, including neurodegenerative and cardiovascular diseases [3,4]. Elevated plasma levels of Hcy have been reported in patients with Alzheimer’s disease; senile, vascular, and other dementias [5]; Parkinson’s disease [6]; cardiovascular disease (CVD) [7]; and heart failure [8,9] and might be a casual factor for disease. Despite large evidence for the involvement of Hcy in these and other diseases, the related pathomechanisms is still not well characterized and are thought to be complex and multifactorial. Numerous experimental studies have shown that Hcy can induce cellular and molecular oxidative injury through reactive oxygen species (ROS) [10,11,12,13,14]. Several mechanisms have been suggested for Hcy-induced oxidative stress, including (i) direct ROS formation via autooxidation in the presence of transition metals, (ii) activation of oxidant systems, and (iii) inhibition of antioxidant systems [15,16,17]. Impairment of epigenetic control mechanisms of gene expression, such as DNA methylation, histone modification, and non-coding RNA, is another possible mechanism of Hcy toxicity [18]. Beyond this, Hcy can change structure and function of proteins by binding to their lysine or cysteine residues; these post-translational modifications (PTMs) are known as N- and S-homocysteinylation, respectively. These mechanisms of Hcy-mediated injury are not mutually exclusive, since altered expression and PTMs of proteins involved in prooxidant/antioxidant pathways can lead to increased cellular oxidative stress and, oppositely, free radicals can induce alterations in gene expression and oxidative PTMs of proteins.

Mitochondria are essential for maintaining cellular homeostasis and function. They play important roles in many cellular processes, including energy production by oxidative phosphorylation, biosynthetic and catabolic pathways, calcium homeostasis, redox homeostasis, and regulation of cell survival and death [19]. Mitochondria are highly dynamic organelles that undergo constant structural and functional remodeling. Dynamic processes such as fusion, fission, movement within the cell, and targeted degradation via mitophagy are essential for maintaining mitochondrial quality and normal cell function [20]. Moreover, regulation of cell function is dependent on communication between mitochondria and other cellular structures, such as the endoplasmic reticulum (ER), plasma membrane, and nucleus [21].

Processes in the brain and heart are critically dependent on mitochondrial function, and a large body of evidence suggests that mitochondrial disorders play an important role in the pathogenesis of the above-mentioned neurodegenerative and cardiovascular diseases [22]. In this review, we discuss the role of hyperhomocysteinemia in mitochondrial dysfunction and focus on ROS as possible mediators of altered cellular functions.

## 2. Homocysteine Transport

Studies on human aortic endothelial cells have shown that Hcy transport across the plasma membrane is mediated by several amino acid transport systems from the solute carrier (SLC) superfamily, including sodium-dependent systems aspartate and glutamate (X_AG_), alanine–serine–cysteine (ASC) and alanine (A), and sodium-independent large neutral branched-chain or aromatic amino acid system L [23,24]. Until now no mitochondrial transporter for HCy in mammalian cells has been identified. Studies on yeast and plant mitochondria have shown that glutamate carriers Ymc2p and BOU can transport L-homocysteine sulfinate but to a much lesser extent than glutamate [25]. Inner mitochondrial membrane contains the S-adenosylmethionine carrier (SAMC), coded by the *SLC25A26* gene. SAMC imports S-adenosylmethionine (SAM) into the mitochondrial matrix, where it is required for methylation reactions. S-adenosylhomocysteine (SAH), produced from SAM after removal of a methyl group, is transported from the matrix to the cytosol by SAMC in exchange for SAM [26]. Studies on cervical cancer cells CaSki have shown that overexpression of *SLC25A26* is associated with accumulation of cytosolic Hcy, increased ROS level, and impaired mitochondrial oxidative phosphorylation [27]. These results suggest that SAMC may play a role in the accumulation of Hcy in mitochondria.

## 3. Homocysteine and Mitochondrial Energy Metabolism

Mitochondria play an essential role in energy production via the electron transport chain (ETC) coupled with oxidative phosphorylation (OXPHOS), the tricarboxylic acid cycle (TCA), and fatty acid β-oxidation. The mitochondrial ETC in mammals is located in the inner mitochondrial membrane and consist of five complexes. Complexes I–IV function as electron carriers. The series of redox reactions provide free energy, which drives pumping of protons into the intermembrane space creating an H^+^ electrochemical gradient–membrane potential (Ψ_m_) across the inner mitochondrial membrane. In turn, this membrane potential is used as a source of energy to synthesize ATP by complex V (ATP synthase). Moreover, membrane potential is also the driving force for mitochondrial Ca^2+^ uptake, which is in physiological conditions essential for regulation of mitochondrial metabolism [28]. However, excessive Ca^2+^ accumulation by mitochondria stimulates the generation of reactive oxygen species (ROS) and leads to the opening of the mitochondrial permeability transition pore (mPTP), which is closed under physiological conditions. Opening of the mPTP is associated with an abrupt increase in the permeability of the inner membrane, membrane depolarization, inhibition of the ETC and ATP synthesis, matrix swelling, and activation of cell death pathways [29]. Alterations in the ETC and OXPHOS and related disturbances in Ca^2+^ homeostasis and ROS production are closely associated with pathological disorders, such as ischemia–reperfusion injury, myocardial infarction, heart failure [30] and neurodegenerative diseases, Parkinson’s disease [31], Alzheimer’s disease [32], and stroke [33]. 

### 3.1. Hcy and Electron Transport Chain

A large body of evidence indicates that HHcy results in decreased mitochondrial respiration associated with reduced activities of ETC complexes and diminished ATP production. One of the first finding was in rat kidney mitochondria, where Hcy inhibited respiration and impaired membrane potential generation, ATP production, and Ca^2+^ handling [34]. In the heart, the effect of Hcy on the function of ETC complexes was evaluated in both HCy-treated tissue slices and animal models of HHcy [35,36,37]. Decreased activities of ETC complexes have been observed, notably of complex II [36,37], complex III [36], and complex IV [35,36,37]. It is well-documented that HCy and its metabolites affect activities of ETC complexes also in brain. Homocysteic acid (HCA) is an oxidized metabolite of Hcy, and administration of Hcy is associated with elevated plasma levels of HCA [38]. Elevated HCA levels were detected, e.g., in hippocampus and cortex of a mice model of Alzheimer’s disease [39]. Seizures induced by bilateral intracerebroventricular infusion of HAC resulted in the inhibition of complex I activity in the cerebral cortex of immature rats [40]. These authors later demonstrated that inhibition of complex I activity persists during the long periods of survival after seizures [41]. The inhibition of complex I was selective, since the activities of complex II and complex IV were unaffected. Recent studies on animal models of chronic HHcy showed inhibition of ETC complexes I, IV, and V in hippocampus [42,43], complex IV in amygdala [44], and complexes IV and V in cerebral cortex [42]. Complex II activity increased while complex IV activity decreased in amygdala of rats after chronic mild HHcy [45]. Studies showing ETC complex I inhibition, motor abnormalities, reduction of striatal dopamine level and degeneration of midbrain dopaminergic neurons in rats after infusion of Hcy into substantia nigra [46], and chronic Hcy treatment of mice [47] suggest involvement of Hcy-related dysfunction of ETC in Parkinson’s disease. There is also evidence that Hcy treatment of the rat ischemic brain inhibits activities of complexes I–III [48]. 

### 3.2. Hcy and Tricarboxylic Acid Cycle

Much less is known regarding the effect of HHcy on TCA, the final common pathway for the oxidation of major nutrients. Activities of citrate synthase and aconitase were measured in rat cortex following seizures induced by homocysteic acid [40,41]. While citrate synthase, which catalyzes the initial reaction of TCA cycle, remained unaffected, aconitase, which is highly susceptible to ROS-induced damage, was inhibited. Decreased activity and reduced protein levels of aconitase and the UQCRC2 component of complex III was demonstrated in Hcy-treated neural stem cells [49]. Overexpression of these proteins by cell transfection with pcDNA-aconitase or pcDNA-UQCRC2 partly restored cell viability reduced by Hcy treatment. Succinate dehydrogenase (SDH), which is the only TCA enzyme bound to the inner mitochondrial membrane, was studied in cardiac mitochondria. While SDH activity was shown to be inhibited in heart slices incubated for 1 h with Hcy [37], activity in rats chronically treated with Hcy increased [35]. Upregulation of isocitrate dehydrogenase (IDH), the key regulatory enzyme of TCA, was shown in hearts of rats with mild HHcy [36], indicating adaptive response to slight increase (~13 μmol/L) in Hcy.

Overall, these studies suggest that HHcy deteriorates mitochondrial energy metabolism by decreasing activities and protein contents of ETC components. However, data on the role of individual components of ETC in Hcy-induced dysfunction are conflicting, and very little is known about effects of HHcy on other mitochondrial components of energy metabolism.

## 4. Homocysteine and Mitochondrial Oxidative Stress

### 4.1. Hcy and Mitochondrial ROS Generation

Using chemiluminescence and fluorescence detection methods the increased mitochondrial ROS generation at elevated Hcy was shown in isolated myocardial mitochondria [35,50], cultured cardiac [51] and neural [49] cells, and heart slices [37]. Significant increases in mitochondrial ROS generation were also observed in brain cortex and hippocampus of rats chronically treated with Hcy [40,42,52]. However, it is worth mentioning that in several studies, the opposite effect of HHcy on heart or brain was observed. Studies on isolated mitochondria showed that Hcy increases ROS production in kidney while ROS production in heart and brain was decreased [53]. Similarly, acute treatment of neuron cells with high concentration of Hcy, which inhibited mitochondrial respiration, reduced the ROS level [54]. Moreover, ROS production and/or oxidative damage was unchanged in brain [45] and heart [36] of rats with chronic mild HHcy. As mentioned earlier, there are several potential mechanisms explaining how Hcy can modulate ROS production, including auto-oxidation of Hcy and altered gene expression and/or post-translational modifications of prooxidant/antioxidant proteins. A number of mechanisms through which Hcy can affect ROS levels and their different significance under distinct conditions may explain these controversial findings.

In the presence of transition metal catalysts, Hcy can generate superoxide radicals (^•^O_2_^–^) and H_2_O_2_ through slow auto-oxidation [55]. Generation of H_2_O_2_ by auto-oxidation of Hcy in the presence of copper has been implicated in injury to cultured endothelial cells and development of atherosclerosis [56]. Although the auto-oxidation of Hcy has been proposed as the mechanism of Hcy-induced ROS generation and mitochondrial dysfunction in the brain [57], direct evidence is lacking. In cardiac mitochondria, generation of superoxide radicals and hydrogen peroxide was induced immediately after initiation of mitochondrial respiration with succinic acid as a substrate [35,50]. This finding suggests that Hcy induces increases in superoxide leak from ETC. Under physiological conditions, leak of superoxide is low and it can activate redox-sensitive signaling pathways, resulting in activation of the antioxidant defense system and low or no oxidative stress. However, an imbalance in ETC can increase mitochondrial ROS concentrations and oxidative stress near the sites of their generation, thus strengthening ETC dysfunction and leading to a vicious cycle of ROS generation. One of the likely mechanisms underlying alterations in activities of ETC complexes and increased ROS leakage under HHcy is protein post-translational modification. Hcy thiolactone, a reactive metabolite of Hcy, can covalently bind to ε-amino group of lysine residues in proteins, and this modification, known as N-homocysteinylation, causes structural and functional changes of proteins [58,59]. Protein N-homocysteinylation was mainly investigated in plasma proteins, but several studies suggest that it contributes to vascular injury [4], impairs neural cell functions [60], increases neurotoxicity of amyloid β-peptide [61], and plays a role in neurodegeneration [62]. To date, a very limited number of studies has attempted to address the issue of N-homocysteinylation of mitochondrial proteins. Cytochrome c is a heme-containing protein bound to the outer leaflet of the inner mitochondrial membrane where it transfers electrons from ETC complex III to complex IV. Four lysine residues of cytochrome c were shown to be susceptible to N-homocysteinylation, and this modification can affect the redox state of protein [63]. Recent spectroscopic studies have shown that homocysteinylated cytochrome c undergoes conformational alterations leading to activation of peroxidase-like activity of this electron carrier [64]. Increased N-homocysteinylation of cytochrome c was observed during cerebral ischemia–reperfusion injury, and this modification seems to play a role in autophagy [65]. Data on N-homocysteinylation of other ETC components are lacking. In contrast to Hcy thiolactone, Hcy itself binds to cysteine residues in proteins, forming stable covalent disulfide bonds. This process, known as S-homocysteinylation, can also potentially alter the structure and function of proteins [66], but its role in mitochondrial injury is unknown. One study that aimed to identify proteins which could be potential targets for S-homocysteinylation due to their structural and physicochemical properties did not reveal any mitochondrial protein [67].

Overall, these findings suggest a complex relationship between HHcy and ROS formation. Current data indicate a crucial role of ETC in mitochondrial ROS; however, it remains unclear which factors determine whether HHcy results in elevated or diminished ROS formation. 

### 4.2. Hcy and Antioxidant Enzymes

Several studies have indicated that oxidative stress at HHcy is linked to an altered antioxidant defense system. As described above, ROS that escape from the ETC are mostly eliminated by antioxidants; however, attenuated antioxidant defense is associated with increased levels of ROS and oxidative stress [68]. The key enzymes that comprise the antioxidant defense system include superoxide dismutases (SODs), catalase, and glutathione peroxidase (GPx). Mitochondrial SOD (MnSOD or SOD2) dismutates superoxide radicals generated in the ETC to H_2_O_2_ and O_2_. Hydrogen peroxide is removed by two types of enzymes, namely glutathione peroxidase (GPx) and catalase, which are less abundant in the brain and heart. Interestingly, chronic HHcy in mice was associated with elevated activity of cytosolic Cu/ZnSOD and catalase in the nucleus caudatus putamen and substantia nigra [47]. Similarly, chronic mild HHcy in rats increased activities of SOD, catalase, and GPx in the amygdala and prefrontal cortex [45]. Increases in antioxidant defense in these tissues was associated with upregulation of Nrf2 (see below) and elimination of ROS production as detected by unchanged 2′,7′-dihydrodichlorofluorescein (DCFH) oxidation. MnSOD activity was shown to be unchanged in the cortex and hippocampus of rats chronically treated with Hcy [42]. In contrast, an imbalance in antioxidant enzymes was observed in the heart. While activity and/or content of MnSOD and catalase were decreased in Hcy-treated groups [35,37], GPx activity was elevated [37]. Moreover, the total antioxidant power was significantly attenuated in H9C2 cardiac cells treated with Hcy [69]. Homocysteine-induced decreases in antioxidant defense were documented in vascular injury [4,70,71,72,73]. Studies on aorta have shown decreased expression of thioredoxin (Trx) and peroxiredoxin (Prx) [74]. In mammalian cells, Trx and Prx are present in the cytosol and different organelles, including mitochondria. They are antioxidants and efficient reductants of protein disulfide bonds, playing a critical role in the control of protein function through their redox state and protein repair [75]. 

Expression of enzyme antioxidants is regulated by the Keap1-Nrf2 signaling pathway, which is recognized as the major regulator of cellular redox homeostasis. Under increased ROS generation, Nrf2 escapes from Keap1-mediated degradation and translocates to the nucleus where it activates transcription of genes encoding antioxidant and detoxification enzymes [76]. Recent studies showed that Nrf2 signaling also plays a role in mitochondrial homeostasis, and Nrf2 function is impaired in mitochondrial disorders [76,77]. Hcy-induced decreases in Nrf2 expression in parallel with reduced expression/activity of antioxidant enzymes and increased ROS production were documented in several studies [52,73,78]. Surprisingly, a recent study has demonstrated that the Nrf2–GPx axis is activated by acute Hcy treatment, while chronic HHcy results in great decline in Nrf2 and GPx7 protein levels [79]. The authors have shown that Hcy activates Nrf2 through oxidation of its inhibitory protein Keap1, while inhibition of Nrf2 pathway under chronic HHcy may result from altered methylation of DNA [79]. Activation of the Nrf2-regulated cascade was confirmed by recent studies on retinal cells. Acute exposure of cultured Müller glial cells to Hcy led to upregulation of Nrf2; increased expression of antioxidant genes, including *catalase*, *Sod2*, and *Gpx1*; and concomitant decreases in ROS and oxidative stress [80]. Further studies from this group have shown that unlike the wild type Müller cells, cells with the deleted Nrf2 gene have significantly reduced basal mitochondrial respiration after exposure to HHcy [81]. Müller cells are the major glial cells in retina, serving as support cells for adjacent neurons. Since HHcy is implicated in the pathogenesis of retinal degenerative diseases, including glaucoma, these data suggest that at least during early stages of HHcy, the protective response is mediated by the Nrf2-regulated antioxidant pathway and mitochondrial energy metabolism. 

Similarly, current data on the role of HHcy in antioxidant defense are controversial. As suggested, diverse mechanisms through which Hcy can modulate the Keap1-Nrf2 pathway under different conditions may result in activation or inhibition of antioxidant enzymes.

### 4.3. Hcy and Hydrogen Sulfide

Catabolism of Hcy via amino acid cysteine is associated with the formation of the small gaseous molecule hydrogen sulfide (H_2_S). H_2_S is a bioactive compound controlling various physiological functions in cardiovascular and cerebrovascular systems [16,82]. It exerts its beneficial effects via vascular smooth muscle cell relaxation, upregulation of antioxidant defense, inhibition of ROS generation, preservation of mitochondrial function, and inhibition of apoptosis [16,83]. Under HHcy, the bioavailability of H_2_S is disturbed; however, controversy exist as to whether the H_2_S level decreases or increases [16,35,84,85]. It has been suggested that a change in the ratio of H_2_S to Hcy is a more valuable parameter than the absolute concentration changes of H_2_S and Hcy levels [85]. Few studies have examined the effect of H_2_S supplementation on mitochondrial functions in HHcy. Protective effects of exogenous H_2_S on Hcy-induced mitochondrial dysfunction were demonstrated in brain. In mitochondria from cortex and hippocampus of hyperhomocysteinic rats, H_2_S supplementation improved mitochondrial respiratory rate, restored Ψ_m_ and prevented apoptosis, restored activities of antioxidant enzymes, decreased mitochondrial ROS formation, and attenuated oxidative damage [42,52,57]. Activation of antioxidant enzymes by H_2_S was mediated via normalization of the Keap1-Nrf2 signaling pathway [52]. Protective effect of H_2_S against Hcy-induced mitochondrial dysfunction and oxidative damage and restoration of MnSOD activity was also demonstrated in cultured brain endothelial cells and heart of hyperhomocysteinic rats [35,86]. Due to the beneficial effects of H_2_S on mitochondrial and cellular functions, H_2_S-releasing compounds are emerging as potential therapeutics for HHcy-associated diseases. The possibilities of H_2_S-releasing therapeutics as well as Hcy-lowering strategies are discussed elsewhere [85].

### 4.4. Hcy and ROS Producing Enzymes

Studies on vascular tissue have also shown that besides suppressing antioxidant defense, Hcy can alter activities of ROS-producing enzymes, such as NADPH oxidase (Nox) and nitric oxide synthase [70,74,86,87], increasing mitochondrial NO and ROS generation [74,86]. Under physiologic conditions, when produced at low levels, NO plays important roles in redox signaling, regulation of mitochondrial function, and cytoprotection [88]. However, when NO and ^•^O_2_^−^ are produced in excess, they react to form peroxynitrite (ONOO^−^), a powerful oxidant that oxidizes aromatic and sulfhydryl group-containing compounds and plays a crucial role in endothelial and vascular dysfunction [89]. On the other hand, overproduction of NO alone was shown to play a beneficial role in HHcy. Increased NO production due to overexpression of inducible NO synthase (iNOS) prevented endothelial cell injury induced by Hcy via S-nitrosylation of Hcy to form S-nitrosohomocysteine (S-NOHcy), which is unable to induce ROS generation and oxidative damage [90]. These findings may at least partly explain the conflicting role of iNOS in CVD [91,92].

### 4.5. Hcy and Expression of Proteins Involved in Energy Metabolism

It is evident from these studies that at least in some tissues, the alterations in antioxidant and prooxidant enzymes may contribute to Hcy-mediated oxidative stress and mitochondrial dysfunction. Some of these studies [37,74,79] also suggest that loss of activity of antioxidant enzymes induced by Hcy may result from altered enzyme content due to altered expression of their genes. S-adenosylhomocysteine (SAH), a precursor of Hcy, is a potent inhibitor of transmethylation reactions dependent on S-adenosylmethionine (SAM) due to reduced SAM/SAH ratio. It has been shown that HHcy is associated with a parallel increase in SAH and a decrease in methylation capacity resulting in hypomethylation of DNA [15,93]. Hcy-mediated hypomethylation of DNA and histone modifications impair epigenetic control of gene expression and may contribute to pathogenesis of various HHcy-related human diseases [18]. Recent comprehensive data mining analyses in human and mouse tissues revealed that 15 nuclear-encoded genes for ETC complex proteins are suppressed in HHcy [94]. Among the identified genes, eleven were of ETC complex I, one of complex IV and two of complex V. Since 4 of the 11 Hcy-suppressed genes of complex I encode core subunits, which are indispensable for activity, the authors suggest that dysfunction of complex I due to alterations in gene expression may play a primary role in Hcy-induced pathogenicity. This study has also shown that heart and brain are among the tissues which are highly responsive to HHcy.

### 4.6. Hcy and Mitochondrial Oxidative Damage

As has been mentioned earlier, imbalance among ETC complexes may result in the increased leakage of ROS and oxidative damage. Substantial evidence for HHcy-associated mitochondrial oxidative damage has been obtained in a variety of tissues in different experimental settings. Mitochondrial DNA (mtDNA) is a double stranded, circular DNA encoding only 13 of all mitochondrial proteins, which are the subunits of ETC complexes I, III, IV, and V. Unlike nuclear DNA, mtDNA is not protected by histone proteins and has less effective repair systems and therefore is more susceptible to oxidative damage and mutations. Numerous diseases have been related to mtDNA mutations, including neurodegenerative diseases, CVD, and aging [95]. Few studies have examined the effects of HHcy on mtDNA. Intermediate HHcy, caused by 4-week folate deprivation, resulted in reduced mtDNA content; the increase of 8-oxodG, a marker of DNA oxidative damage; and an increase in the frequency of mtDNA deletions in various rat tissues, including brain, heart, and liver [96]. Interestingly, in the liver tissue the increases in expression of mRNA for nuclear-encoded and mitochondrial-encoded subunits of complex IV and cytochrome c were observed. This suggests that an adaptive mechanism is activated in some tissues to compensate mtDNA damage. Methionine restriction, associated with a decrease in Hcy, resulted in decreases in mitochondrial ROS production and oxidative damage to mtDNA detected by 8-oxodG [97]. 

Due to their abundance in cells, proteins are likely to be primary targets of ROS. Protein modifications, such as sulfhydryl group oxidation, tyrosine nitration, and carbonyl formation, have been described in heart [37], brain [41], and vascular tissues [98] under HHcy. Generally, oxidative modifications of proteins result in structural changes and a loss of function. Despite strong evidence that oxidative damage to mitochondrial proteins is involved in HHcy-induced pathogenesis, oxidatively-modified proteins were not yet identified.

## 5. Homocysteine and Mitochondrial Apoptotic Pathway

ROS and mitochondrial damage promote the intrinsic, also called mitochondrial, apoptotic pathway. Decreases in membrane potential and ATP content and increased mitochondrial membrane permeability allow for translocation of pro-apoptic factors, such as cytochrome c, apoptosis inducing factor (AIF), and Smac/DIABLO, from mitochondria to cytosol, leading to the activation of the caspase signaling pathway and apoptosis. The mitochondrial apoptotic pathway is regulated by the ratio of pro-apoptotic and anti-apoptotic proteins of the B-cell lymphoma protein 2 (Bcl-2) family. Pore forming pro-apoptotic Bcl-2 proteins, Bak and Bax, can induce permeabilization of the outer mitochondrial membrane, depolarization of the inner membrane, and release of pro-apoptic factors. In viable healthy cells, the pore forming pro-apoptotic proteins are neutralized by anti-apoptic proteins of the Bcl-2 family, such as Bcl-2 or Bcl-xl. In response to multiple types of cellular stress, pro-apoptic proteins of the Bcl-2 family, also termed BH3 domain-only proteins, such as PUMA, Bad, or Noxa, are activated at different levels (e.g., transcription or post-translation modifications). The activation results in binding of BH3 domain-only proteins to anti-apoptotic proteins followed by Bax and/or Bak activation and consequent initiation of mitochondrial apoptosis [99].

### 5.1. Hcy and Pro-Apoptic and Anti-Apoptic Proteins

Results from isolated H9c2 cardiomyocytes revealed that the effect of Hcy on cell viability is concentration-dependent [100]. The lower concentration of Hcy (0.1 mmol/L) resulted in an increase in Ψ_m_ and ATP concentration. The intermediate concentration of 1.1 mmol/L Hcy caused morphological alterations of mitochondria with slight decreases in Ψ_m_ and ATP content. In cells incubated with 2.7 mmol/L Hcy, further decreases in ΔΨ_m_ and ATP content occurred, leading to apoptosis and necrosis. Recent studies on H9c2 cells by other authors have confirmed these findings, showing that HHcy results in up-regulation of Bad and Bax and down-regulation of Bcl-2 [51,69]. Several experimental studies have shown that also in neuronal and vascular cells, HHcy induces apoptosis via disturbed mitochondrial homeostasis and oxidative stress [57]. Decreased mitochondrial membrane potential, upregulation of pro-apoptotic and downregulation of anti-apoptotic proteins, release of cytochrome c, or activation of caspase-9 and its downstream caspase-3 was shown in different cell lines, including hippocampal neuronal cells [78,101,102], cerebellar granule cells [103], neuroblastoma cells [57,104,105], brain endothelial cells [86,106], human umbilical vein endothelial cells (HUVECs) [69,72,73,107,108,109], heart microvascular endothelial cells [110], aortic endothelial cells [111], as well as in animal models [112,113]. Studies on hippocampal neuronal cells [101] suggest that DNA damage and activation of poly-ADP-ribose polymerase (PARP), involved in DNA repair, are the early events in the Hcy-induced neurotoxicity required for the subsequent mitochondrial ROS generation and decline in inner mitochondrial membrane potential. Hcy-induced neuronal cell death is thought to be mediated via the activation of glutamate N-methyl-D-aspartate receptors (NMDARs) [114,115]. Earlier studies on cerebellar granule cells showed that mitochondrial alterations play a similar role in acute HHcy and glutamate-induced neurotoxicity, but HHcy toxicity is less dependent on calcium imbalance [103]. Later studies confirmed that Hcy-mediated cell death is different from glutamate NMDAR-induced excitotoxic cell death and showed that it involves a sustained low-level increase in intracellular Ca^2+^ and subsequent activation of the extracellular signal-regulated kinase-mitogen-activated protein (ERK-MAP) kinase pathway [116,117,118]. NMDR-mediated Ca^2+^ accumulation was also shown in Hcy-treated brain endothelial cells, and disturbed Ca^2+^ homeostasis seems to play a role in decreased mitochondrial membrane potential and mitochondrial dysfunction [86]. Studies on hippocampal neurons suggest that Hcy-induced excessive Ca^2+^ influx activates calcineurin, calcium/calmodulin-dependent protein phosphatase. Calcineurin dephosphorylates pro-apoptic Bad to stimulate its translocation from cytosol to mitochondria and induction of apoptosis [102]. Of note, studies on vascular cells (HUVECs) suggest that besides activating the mitochondrial apoptotic pathway, Hcy can stimulate cell death by inhibiting the PI3K/Akt/eNOS signaling pathway, which is involved in prevention of apoptosis [73,119]. 

A key role in the control of apoptosis is played by the p53 protein. After activation by a variety of stress factors, p53 controls both intrinsic and extrinsic apoptic pathways at multiple steps, including expression of pro- and anti-apoptic proteins [120]. In HUVECs, Hcy-induced upregulation of p53 resulted in increased expression of proapoptic Noxa protein and apoptosis [90]. Noxa is a proapoptic BH3 domain-only protein, a member of the Bcl-2 family, which promotes permeabilization of the outer mitochondrial membrane, the commitment step to cell death via the intrinsic apoptic pathway. Besides the BH3 domain, the Noxa protein also has a mitochondrial targeting domain (MTD). The BH3 domain is responsible for inducing apoptosis; however, recent studies have shown that MTD of Noxa can cause necrotic cell death by opening mPTP through voltage-dependent anion channels (VDACs) of the outer mitochondrial membrane [121]. Moreover, MTD was shown to cause endoplasmic reticulum (ER) damage, indicating the role of endoplasmic reticulum (ER) stress in Noxa-induced cell death [122].

### 5.2. Hcy and Mitochondria-ER Crosstalk

ER and mitochondria are joined together with a specialized set of redox-sensitive proteins at specific domains, termed mitochondria-associated membranes (MAMs) [123,124]. Mitochondria–ER contacts play important roles in various functions, such as Ca^2+^ handling, redox signaling, apoptosis, ER stress, and regulation of mitochondrial morphology. ER stress is an important factor in mitochondrial dysfunction. ER is the major intracellular source of calcium, and excessive Ca^2^^+^ release from ER, induced, e.g., by mitochondrial ROS, causes Ca^2^^+^ overload in mitochondria and triggers apoptosis. The RNA-dependent protein kinase (PKR)-like ER kinase (PERK) is an ER-transmembrane protein and a key sensor of ER stress. Sustained activation of PERK caused by severe or persistent ER stress results in upregulation of a pro-apoptic transcription factor C/EBP homologous protein (CHOP), and activation of nuclear factor kappaB (NF-κB), triggering the apoptotic pathway [125]. Involvement of mitochondria–ER crosstalk in Hcy-induced apoptosis was shown in HUVECs [21,87]. Prolonged treatment of HUVECs with Hcy increased expression of Nox4, which is expressed primarily in mitochondria, activated PERK, upregulated CHOP, activated NF-κB, and induced apoptosis. Inhibition of Nox4 resulted in decreased formation of ROS and alleviated apoptosis and ER stress. Moreover, inhibition of the PERK pathway partly alleviated Hcy-induced apoptosis. The 78-kDa glucose-regulated protein GRP78, also known as BiP, is the major ER chaperone, which plays a critical role in cell survival and death. Grp78 is activated after ER stress as part of unfolded protein response (UPR), which is regulated by several signaling pathway, including PERK [126,127]. Several studies on myocardial, brain, and endothelial tissues or cells have shown that HHcy is associated with upregulated expression of Grp78 and other proteins involved in mitochondria–ER crosstalk and apoptosis, such as CHOP, caspase 12, p53 up-regulated modulator of apoptosis (PUMA), 60 kDa heat shock protein, and VDAC [35,36,70,87,105,128,129]. As mentioned earlier, mitochondria–ER crosstalk can modulate mitochondrial morphology. Swollen mitochondria with fractured and dissolved cristae and a dilated ER membrane network were observed in methionine overload-induced HHcy in rats as well as in H9C2 cardiac cells treated with Hcy [128]. Similar alterations in the ultrastructure of mitochondria and ER occurred in cells treated with thapsigargin or tunicamycin, inducers of ER stress, indicating the role of Hcy-induced ER stress in mitochondrial damage. Interestingly, H_2_S supplementation decreased expression of ER stress-related proteins and attenuated Hcy-induced ER stress [128].

### 5.3. Hcy, Inflammation and Apoptosis

Inflammation plays an important role in vascular disease and hypertension. Studies of Tyagi’s group have shown that HHcy promotes vascular inflammation via activation of Toll-like receptor 4 (TLR-4), leading to initiation of mitochondrial apoptotic cell death. Mutations of TLR-4 attenuated chronic vascular inflammation and mitochondrial dysfunction in hyperhomocysteinic mice [113,130].

## 6. Homocysteine and Mitochondrial Dynamics

Mitochondrial dynamics is an important mechanism of regulation of mitochondrial morphology and function. This term encompasses mitochondrial fusion, fission, transportation, and selective degradation—mitophagy [20,131]. In mammalian cells, mitochondrial fusion is controlled by mitofusins 1 and 2 (Mfn 1 and Mfn2, respectively) located in the outer mitochondrial membrane, and optic atrophy 1 (Opa1) protein, which regulates fusion of inner membranes. Mitochondrial fission is mediated by cytosolic GTPase dynamin-related protein (DRP1), which binds to outer membrane receptors, like Fis 1, to constrict both outer and inner membranes and divide the mitochondrion into two daughter organelles [20,131]. By fission, a daughter mitochondrion, which may contain deleterious or damaged components, is removed via mitophagy. In mouse brain endothelial cells, Hcy upregulated DRP1, Mfn2, and autophagy marker LC-3 [106]. Studies on retinal ganglion neurons show Hcy-induced disbalance of mitochondrial fusion and fission. Using cystathione-β-synthase-deficient mice as a model of moderate HHcy, increased expression of Opa 1 and Fis 1 proteins in retinal ganglion neurons was demonstrated [132]. The alterations in Opa 1 and Fis 1 levels were associated with increased mitochondrial fission as suggested by the increased number of small mitochondria [132]. HHcy-induced mitophagy was demonstrated in cardiac mitochondria [133]. NMDA receptor 1 is present not only in mammalian neurons but also in cardiomyocytes and endothelial cells, and as mentioned earlier, has affinity to Hcy. Studies on mice with cardiac-specific deletion of this receptor suggest that Hcy activates mitochondrial matrix metalloproteinase-9 (mtMMP-9) and induces translocation of connexin-43 (Cxn-43) to the mitochondria, resulting in mitophagy [133]. 

## 7. Conclusions

**Figure 2:**
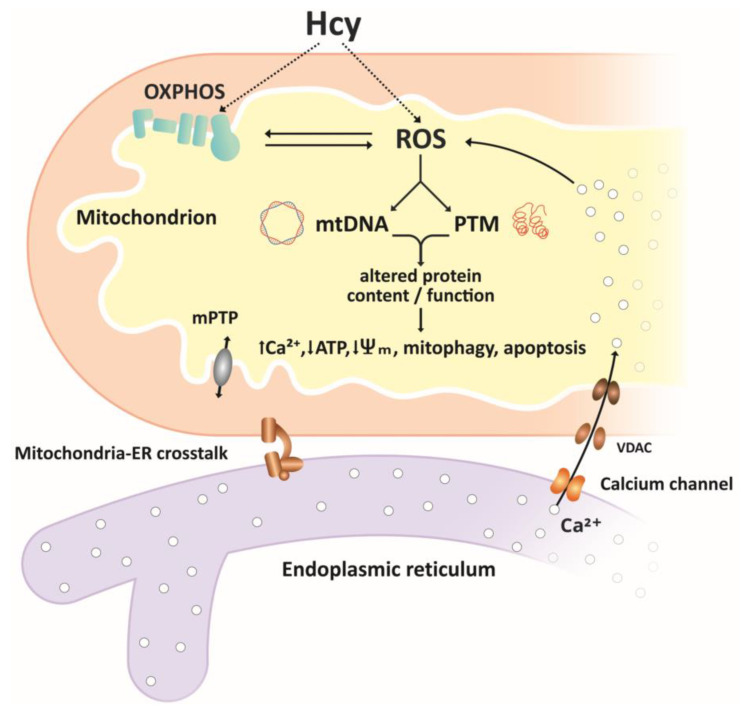
Simplified overview of homocysteine effects on mitochondria. Reactive oxygen species ROS, produced in the electron transport chain ETC or through autooxidation of Hcy, are important mediators of Hcy effects on mitochondrial function. ROS-associated oxidative damage to mtDNA and post-translational modifications PTMs of proteins result in altered content and/or function of mitochondrial proteins, including components of the ETC, antioxidant/pro-oxidant enzymes, membrane carriers, and receptors. Altered function of mitochondrial proteins may result in further increases in ROS levels, accumulation of Ca^2+^ ions mediated by mitochondria–ER crosstalk, decrease in ATP production, decreased membrane potential, and initiation of mitophagy and apoptosis.

Mitochondria fulfill many essential cellular functions, and mitochondrial disorders are associated with development of various neurological and cardiovascular diseases. The evidence summarized in the present review indicates that homocysteine, a widely accepted risk factor for these diseases, affects normal mitochondrial structure and function, including energy production, mitochondrial dynamics, and cell survival and death (Figure 2). Currently available data provide evidence that ROS are important mediators of Hcy injury. However, detailed molecular links between Hcy-induced oxidative stress and mitochondrial dysfunction are not fully understood, and several questions remain unanswered. Although ETC appears to be a major source of ROS under HHcy, individual ROS target-modified biomolecules were not yet identified. Several studies reviewed in this article suggest that HHcy might be not only detrimental but also beneficial for mitochondrial ROS homeostasis and cell viability. The reason for this discrepancy might be related to multiple pathways through which Hcy can modify cell functions and their diverse impact in different conditions and/or tissues. Future research should define conditions at which HHcy stimulates protective mechanisms, activates gene expression, ameliorates antioxidant defense, and reduces mitochondrial oxidative stress. Better knowledge about the mechanisms that mediate effects of Hcy on mitochondria might be helpful for the development of more specific therapeutic strategies for neurological and cardiovascular diseases.

## Abbreviations

CVD	cardiovascular disease
DCFH	2′,7′-dihydrodichlorofluorescein
DRP1	dynamin-related protein
ER	endoplasmic reticulum
ETC	electron transport chain
GPx	glutathione peroxidase
Hcy	homocysteine
HHcy	hyperhomocysteinemia
HUVECs	human umbilical vein endothelial cells
MAMs	mitochondria-associated membranes
mtDNA	mitochondrial DNA
mPTP	mitochondrial permeability transition pore
MTD	mitochondrial targeting domain
NMDAR	N-methyl-D-aspartate receptor
NOS	NO synthase
OXPHOS	oxidative phosphorylation
PTMs	post-translational modifications
ROS	reactive oxygen species
SAH	S-adenosylhomocysteine
SAM	S-adenosylmethionine
SOD	superoxide dismutase
TCA	tricarboxylic acid cycle
VDAC	voltage-dependent anion channel
